# Impact of Mitochondrial Genetic Variants in *ND1*, *ND2*, *ND5*, and *ND6* Genes on Sperm Motility and Intracytoplasmic Sperm Injection (ICSI) Outcomes

**DOI:** 10.1007/s43032-020-00449-3

**Published:** 2021-01-21

**Authors:** Mohammad A. Al Smadi, Mohamad Eid Hammadeh, Erich Solomayer, Osamah Batiha, Mohammad M. Altalib, Mohammad Y. Jahmani, Mohammad A. Shboul, Bassam Nusair, Houda Amor

**Affiliations:** 1 Department of Obstetrics & Gynecology, Reproductive Medicine Unit, Saarland University, Homburg, Germany; 2 Department of Biotechnology & Genetic Engineering, Jordan University of Science and Technology, Irbid, Jordan; 3 Department of Statistics, Yarmouk University, Irbid, Jordan; 4 Department of Medical Laboratory Sciences, Jordan University of Science and Technology, Irbid, Jordan; 5 Reproductive Endocrinology and IVF Unit, King Hussein Medical Centre, Amman, Jordan

**Keywords:** mtDNA, Asthenozoospermia, Sperm motility, Fertilization rate, Embryo cleavage score, Embryo quality score

## Abstract

Sperm mitochondrial dysfunction causes the generation of an insufficient amount of energy needed for sperm motility. This will affect sperm fertilization capacity, and thus, most asthenozoospermic men usually require assisted reproductive techniques. The etiology of asthenozoospermia remains largely unknown. The current study aimed to investigate the effect of mitochondrial genetic variants on sperm motility and intracytoplasmic sperm injection (ICSI) outcomes. A total of 150 couples from the ICSI cycle were enrolled in this study. One hundred five of the male partners were asthenozoospermic patients, and they were subdivided into three groups according to their percentage of sperm motility, while forty-five of the male partners were normozoospermic. Genetic variants were screened using direct Sanger’s sequencing in four mitochondrial genes (nicotinamide adenine dinucleotide hydrogen (NADH) dehydrogenase 1 (ND1), NADH dehydrogenase 2 (ND2), NADH dehydrogenase 5 (ND5), and NADH dehydrogenase 6 (ND6)). We identified three significant variants: 13708G>A (rs28359178) in *ND5*, 4216T>C (rs1599988) in *ND1*, and a novel 12506T>A in *ND5* with *P* values 0.006, 0.036, and 0.013, respectively. The medians of sperm motility, fertilization rate, embryo cleavage score, and embryo quality score were significantly different between men showing 4216T>C, 12506T>A, 13708G>A and wild type, Mann-Whitney *P* values for the differences in the medians were < 0.05 in all of them. The results from this study suggest that 13708G>A, 12506T>A, and 4216 T>C variants in sperm mitochondrial DNA negatively affect sperm motility and ICSI outcomes.

## Introduction

Genetic variants in mitochondrial genes are associated with many diseases [1]. Most of these diseases affect organs with high energy demand, such as the brain, the skeletal muscle, the eye, and the heart [2]. Because mitochondrial DNA (mtDNA) is not protected by histones or other DNA-binding proteins, it is more prone to DNA damage caused by excess levels of reactive oxygen species (ROS) and free radicals present in the matrix [3]. Furthermore, the mtDNA repair mechanism is less efficient compared to nuclear DNA repair [4]. Together, these factors increase the mutation rate in mtDNA, compared to nuclear DNA by 10–100 times [5].

Sperm depends on the mitochondrial oxidative phosphorylation (OxPhos) mechanism to produce the required energy for its motility [6]; therefore, mutated mtDNA leads to energy insufficiency, which causes a reduction in sperm motility [7]. In comparison to the oocyte, which has around 150,000 mtDNA copies, the spermatozoa have only around 100 mtDNA copies [8].

Sperms usually produce ROS in a natural physiological process, and ROS at low levels are necessary for sperm function, as they play a significant role in sperm maturation, acrosome reaction, and sperm-oocyte fusion [9]. Excess levels of ROS have a damaging effect, leading to nuclear DNA strand breaks [10]. Leukocytes in the semen produce ROS one thousand times more than sperms, where such a situation is common during urinary tract infection (UTI) [11]. Mitochondrial dysfunction, caused by the production of high levels of ROS from complex I, has been shown to cause lipid peroxidative damage to the sperm midpiece and to result in a reduction in the sperm motility [12].

Seminal plasma is the major source of antioxidants that protect sperm cells against oxidative damages [13]. A significant decrease in the reduced to oxidized glutathione ratio and total glutathione levels in seminal plasma among groups of males with varicocele and idiopathic infertility has been reported [14].

It has been thought for a long time that mtDNA was inherited restrictedly from the mother, but a recent study has challenged this idea and provided evidence of additional paternal mtDNA transmission from fathers to offspring [15], where paternal mtDNA is governed by a quasi-Mendelian inheritance [16]. Furthermore, during intracytoplasmic sperm injection (ICSI) the whole sperm is injected into the cytoplasm of the oocyte, interestingly, the mtDNA is occasionally preserved, so that the offspring may indeed share their father’s mitochondrial DNA [17].

Sperm motility has been shown to be affected by variations in the mitochondrial genome. Two single nucleotide polymorphisms (SNPs) in *ATPase6* and *ND4* mitochondrial genes at 9055 and 11719 loci, respectively, were found to be associated with asthenozoospermia [18]. Furthermore, a recent study has found a missense variant (11696G>A) in the *MT-ND4* gene to be associated with reduced sperm motility and causing the replacement of valine residue at position 313 with isoleucine, leading to a change in the secondary structure of the protein [19].

Several studies have shown connections between mitochondrial mutations, sperm motility, fertilization rate, and pregnancy completion. A study found that the fertilization rate had a strong positive correlation with sperm motility [20]. Moreover, a point mutation in the *ND1* gene at locus 4216 has been associated with recurrent pregnancy loss [21]. On the other hand, another study found that embryo grading at day 3 can predict the pregnancy rate of the in vitro fertilization (IVF) cycle, where embryos with high-quality scores have a better chance of a successful embryo transfer compared to embryos with a low-quality score [22].

The aim of this study was to investigate the influence of genetic variations in four mitochondrial genes (*ND1*, *ND2*, *ND5*, and *ND6*) on sperm motility and ICSI outcomes. This study is part of a larger project that aims to understand the role of mitochondrial genetic variants in infertility.

## Methods

### Subjects

From August 2018 to October 2019, samples were collected from 150 male partners, aged < 40 years, of ICSI couples that attended Assisted Reproductive Techniques (ART) Department at Prince Rashid Bin AL Hassan Hospital (PRBH), Irbid, Jordan.

According to the World Health Organization (WHO) laboratory manual for semen analysis, the total motility (progressive motility (PR) and non-progressive motility (NP)) for a normal male should be above 40%; otherwise, it will be considered to be asthenozoospermic. One hundred five of these samples were from asthenozoospermic men (PR + NP < 40%) and were divided into three groups according to their percentage of sperm motility. Group 1 included patients with sperm motility from 0 to 5%, group 2 included patients with sperm motility from 6 to 15%, and group 3 included patients with sperm motility from 16 to 35%. Forty-five samples of normozoospermic men with a high percentage of sperm motility between 50% and 75% were also collected as controls. The other semen parameters were within normal ranges (Table 1), Patients with varicocele, and alcoholic problems, as well as cigarette smokers and patients with genetic abnormalities, such as Klinefelter’s syndrome, were excluded from this study. The study was approved by the Jordanian Royal Medical Services-Human Research Ethics Committee on 30/7/2018 with the project identification code (TF3/1/Ethics Committee/9126), and written consent from each couple was obtained.

**Table 1:** Semen parameters among groups

	Semen volume (ml), median ± SD	Sperm concentration (10^6^ per ml), median ± SD	Total motility (PR + NP %), median ± SD	Morphologically normal spermatozoa (%), median ± SD
Group 1	2.63 ± 1.18	32.04 ± 15.12	0 ± 2.57	5.78 ± 3.14
Group 2	3.1 ± 2.16	40.56 ± 27.88	9 ± 3.40	4.89 ± 2.45
Group 3	2.89 ± 1.43	61.13 ± 39.74	20 ± 6.99	6.13 ± 5.69
Control	3.2 ± 1.35	73.16 ± 52.21	58 ± 9.05	8.82 ± 7.03

Semen samples were obtained from all subjects by masturbation after 3 to 5 days of sexual restraint. The samples were incubated at 37 °C for 30 min (min) to allow liquefaction. Then they were evaluated by a senior clinical embryologist according to WHO criteria (WHO, 2010).

### Semen Preparation for ICSI

Semen samples were fractionated by Percoll media (45% and 90% gradient) through centrifugation at 1000*g* for 22 min. After that, the pellet was collected and washed twice with a sperm-washing medium. Supernatants were discarded, and then the pellet was gently layered with 4-(2-hydroxyethyl)-1-piperazineethanesulfonic acid (HEPES) (21 mM, PH = 7.3) plus 0.5% human serum albumin (Sage, USA). After that, the sperm pellet was collected and placed in the CO_2_ incubator at 37 °C for 1 h, and later on the surface layer was aspired. Only sperm samples with 0% percentage of sperm motility were washed without this HEPES layering.

### The ICSI Technique

The oocytes were denudated after 2 h of egg retrieval by both chemical and mechanical treatments. Chemical denudation was performed using a hyaluronidase enzyme, while the mechanical denudation was done by aspirating the oocytes through glass pipettes (with a 150–300 μm inner diameter) in HEPES, covered with oil (Vitrolife, Sweden). After 3 h in the CO_2_ incubator at 37 °C, only the mature metaphase II oocytes were selected for injection by ICSI, using the microscope Integra 3 micromanipulator (CooperSurgical Fertility Company, Denmark) [23].

### Fertilization and Embryo Assessment

Zygotes were evaluated and graded from 1 to 5 after 16–18 h post-ICSI, and embryos at day 3 were classified as grades A, B, C, and D according to the Scott scoring system [24]. The cleavage score of each patient was calculated as follows: the sum of cleavage scores of embryos/the total number of embryos, where embryos at day 3 with 8 cells were given 4 points, embryos with 6 cells were given 3 points, and embryos with 4 cells were given 2. The cumulative quality score of the embryos for each patient was calculated as follows: the sum of scores of embryos/the total number of embryos, where embryos with grade A were given 3 points, embryos with grade B were given 2 points, and embryos with grade C were given only 1 point [25].

### Sperm mtDNA Extraction

Genomic DNA was extracted from the purified samples using a commercial kit (QIAamp DNA Mini Kit, Qiagen, Germany); then, mtDNA was amplified using the REPLI-g Mitochondrial DNA Kit (Qiagen, Germany). Using the Nanodrop spectrophotometer ND-2000c (Thermo Scientific, USA), only the isolated DNA with an optimal density ratio of 260/280 of 1.8 or more was chosen and stored at − 20 °C.

### PCR

To amplify the *ND1, ND2, ND5 and ND6* genes, 4 sets of polymerase chain reaction (PCR) primers (forward and reverse) were designed using the Primer 3 program, flanking the region of each gene. Primers were designed using the human mitochondrial sequence obtained from the National Centre of Biotechnology Information (NCBI) (http://www.ncbi.nlm.nih.gov). The oligonucleotide primers were synthesized by Microsynth Seqlab in Germany (Table 2).

**Table 2:** Primers list for PCR amplification and Sanger sequencing

Primer name	Sequence (5′– 3′)	Product length
MT-ND1-F	CACCCACCCAAGAACAGGGT	1155
MT-ND1-R	TTCTCAGGGATGGGTTCGATTC	
MT-ND2-F	TCAGCTAAATAAGCTATCGGGC	1200
MT-ND2-R	GAGTGGGGTTTTGCAGTCCT	
MT-ND5-F	CTGCTAACTCATGCCCCCAT	2043
MT-ND5-R	GGAGGATCCTATTGGTGCGG	
MT-ND6-F	CCTCTCTTTCTTCTTCCCACTCA	622
MT-ND6-R	CGATGGTTTTTCATATCATTGGTCG	
ND5A^*^	CTAAACGCTAATCCAAGCC	*
ND5B^*^	CTATTACTCTCATCGCTACCTC	*

ND5A^*^ and ND5B^*^ are additional internal primers were designed for Sanger sequencing only

A 25 μL reaction mixture was prepared to contain 12.5 μL PCR Master Mix (2X) (Thermo Scientific), 0.8 μL of 10 mM forward primer, 0.8 μL of 10 mM reverse primer, 2 μL mtDNA (20 ng/μL) and 8.9 μL nuclease-free water. The Thermocycler (C1000™ Thermal cycler, Bio-Rad, USA) program was set as follows: initial denaturing at 95 °C for 3 min, followed by 35 cycles of denaturation at 95 °C for 30 s, annealing for 40 s (*ND1*: 59 °C; *ND2* and *ND6*: 61 °C; *ND5*: 64 °C), an extension of primers at 72 °C for *ND1* and *ND2*: 1 min; *ND5*: 2 min; *ND6*: 45 s), then a final extension for 5 min at 72 °C. To check the amplification, 5 μL of each PCR product was run on 1% agarose gel stained with SYBR Safe stain (Invitrogen) and then visualized using Molecular Imager Gel Doc XR+ (Bio-Rad).

### Identification of Genetic Variants in *ND1*, *ND2*, *ND5*, and *ND6*

PCR products were purified and sequenced using the Sanger method (Microsynth Seqlab, Germany). Sequencing was carried out in both directions (forward and reverse) for each sample. For the *ND5* gene, two additional internal primers were designed, namely, ND5A and ND5B (Table 1).

The primary and secondary sequences for each sample were analyzed using the BioEdit sequence alignment editor version 7.2.5 and aligned to the NCBI reference sequences (NC_012920.1).

To predict the possible impact of amino-acid substitution on protein structure and function, and to evaluate the possible damaging effect of genetic variants; two versions of software were used (The American College of Medical Genetics and Genomics (ACMG),https://www.acmg.net and Poly Phenyl-2, http://genetics.bwh.harvard.edu/pph2.

### Statistical Analysis

Statistical analysis was carried out using the OriginPro, Version 2020 (OriginLab Corporation, Northampton, MA, USA). The normality assumptions were checked for the variables in question and were found not to be fitted by a normal distribution, and hence, non-parametric tests were applied to our study. The Kruskal-Wallis H test alongside the Mann-Whitney *U* test in addition to chi-square were used to determine if there were statistically significant differences between two or more groups of an independent variable on a continuous or ordinal dependent variable. Spearman’s rho, a non-parametric test, was used to measure the strength of association between two variables. Odds ratio and their 95% confidence intervals were determined, and some descriptive statistics and graphs for the variables in question were presented. A *P* value < 0.05 was considered to be statistically significant.

## Results

**Fig. 1:**
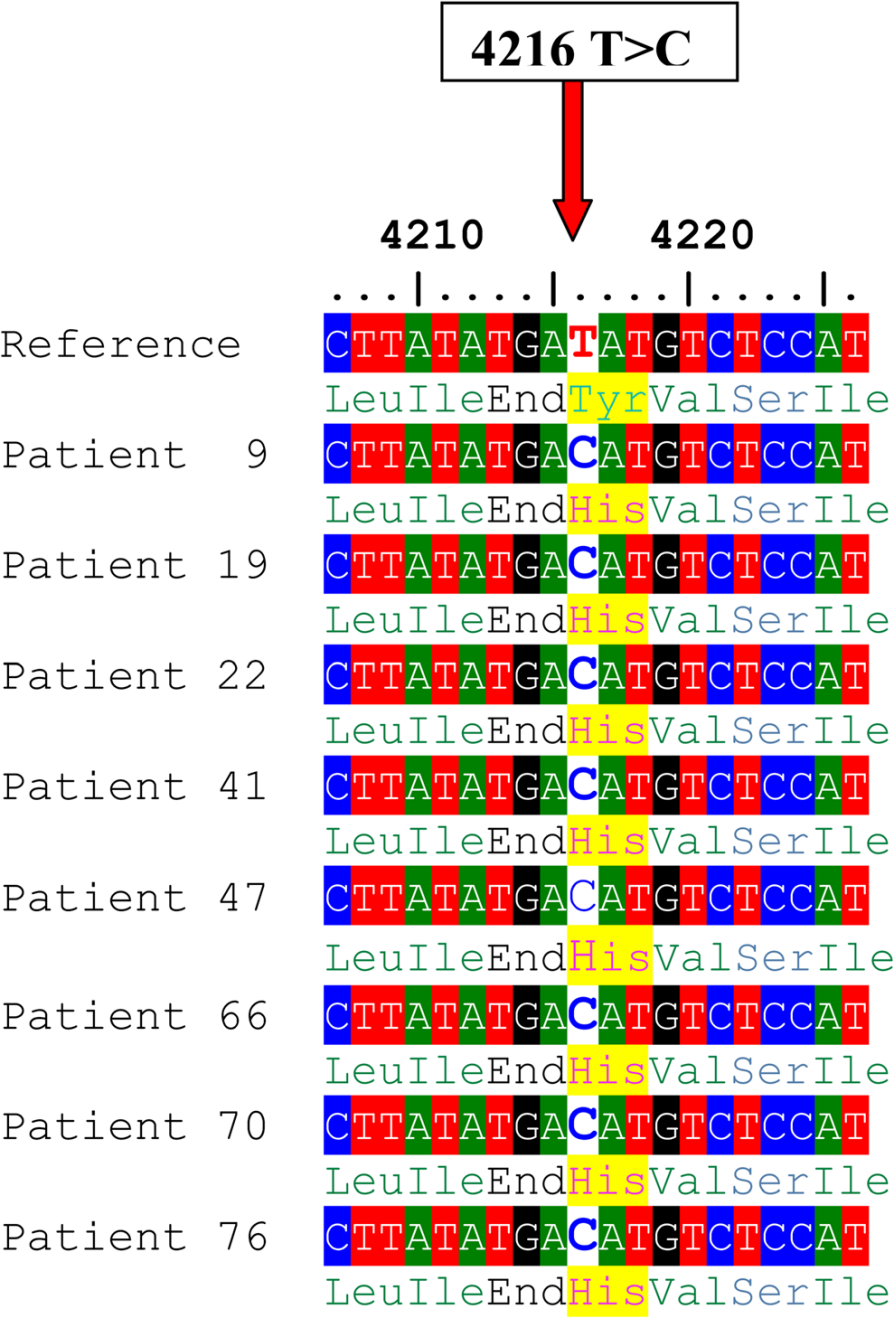
Alignment of the ND1 gene sequence for 8 patients showing 4216T>C nucleotide substitution. The red arrow indicates the site of nucleotide substitution, and the highlighted yellow colour indicates the amino-acid replacement (Tyr>His)

**Fig. 2:**
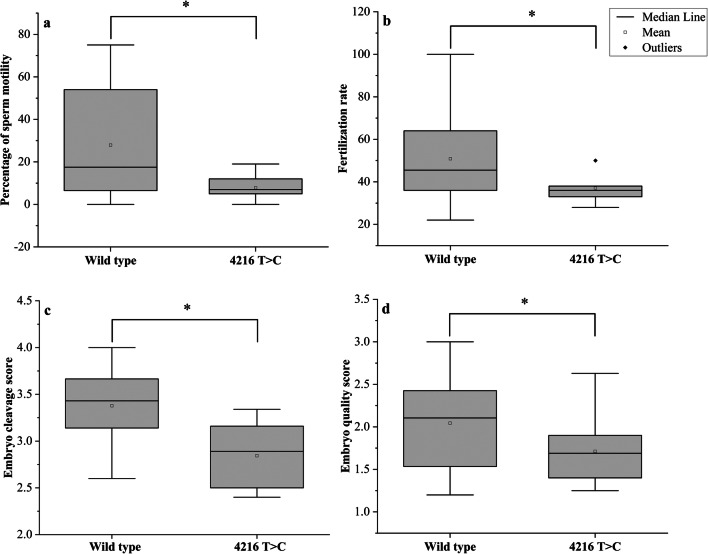
Box plots showing the differences between men with or without the 4216T>C. **a** Difference in sperm motility, **b** difference in fertilization rate, **c** difference in embryo cleavage score, and **d** difference in embryo quality score. Mann-Whitney *P* values for the differences in the medians were 0.012, 0.013, 0.044, and 0.041, respectively. *P < 0.05

A total of 29 nucleotide substitutions (SNPs) in the *ND1* gene were identified; six of them were missense, while 23 were synonymous (Table 3). The percentages of men with total variants in the *ND1* gene among groups 1, 2, 3, and the control were: 80%, 25.7%, 17.1%, and 15.6%, respectively, *P* = 0.0001(Table 4). Only one variant 4216T>C (rs1599988) was significantly different between cases and controls (*P* value = 0.006). The percentages of men with 4216T>C among groups 1, 2, and 3 were 14.3%, 8.6%, and 5.8%, respectively (*P* = 0.005), while it was not found among the control. This variant caused Tyr>His amino acid substitution (Fig. 1). According to ACMG and Poly Phenyl-2, it is predicted that this variant is benign (score 0.001, sensitivity 0.99; specificity 0.15). It was identified among 10 asthenozoospermic patients in a homoplasmic state while it was not found among control group. The medians of sperm motility were (wild type (17.5 ± 25.21), 4216T>C (7 ± 6.1), *P* = 0.012), fertilization rate (wild type (45.5 ± 18.08, 4216T>C (36 ± 7.61), *P* = 0.013), embryo cleavage score (wild type (3.42 ± 0.37), 4216T>C (3.07 ± 0.12), *P* = 0.044), and embryo quality score (wild type (2.11 ± 0.49), 4216T>C (1.69 ± 0.41), *P* = 0.041) (Fig. 2).

**Table 3:** The mtDNA variants identified in the *ND1* gene

Serial Number	MtDNA variant	Amino Acid change	Frequency of variant in control	Frequency of variant in asthenozoospermia	G test	*P* value
1	3316G>A	Ala>Thr	0/45	5/105	3.64	0.056
2	3348A>G	Leu>Leu	2/45	3/105	0.234	0.629
3	3480A>G	Lys>Lys	2/45	6/105	0.104	0.747
4	3462C>T	Ala>Ala	0/45	3/105	2.166	0.141
5	3537A>G	Leu>Leu	0/45	1/105	0.716	0.397
6	3594C>T	Val>Val	1/45	3/105	0.051	0.822
7	3720A>G	Gln>Gln	0/45	1/105	0.716	0.397
8	3741C>T	Thr>Thr	0/45	1/105	0.716	0.397
9	3826T>C	Leu>Leu	0/45	1/105	0.716	0.397
10	3882G>A	Gln>Gln	0/45	2/105	1.438	0.23
11	3921C>T	Ser>Ser	0/45	1/105	0.716	0.397
12	4086C>T	Val>Val	0/45	1/105	0.716	0.397
13	4216T>C^*^	Tyr>His	**0/45**	**10/105**	**7.436**	0.006^*^
14	4017C>T	Leu>Leu	0/45	1/105	0.716	0.397
15	3705G>A	Leu>Leu	0/45	3/105	2.166	0.141
16	3505A>G	Thr>Ala	0/45	1/105	0.716	0.397
17	4104A>G	Leu>Leu	1/45	4/105	0.266	0.606
18	3847T>C	Leu>Leu	0/45	1/105	0.716	0.397
19	3834G>A	Leu>Leu	1/45	1/105	0.354	0.552
20	3843A>G	Trp>Trp	0/45	1/105	0.716	0.397
21	3819C>T	His>His	0/45	1/105	0.716	0.397
22	3335T>C	Ile>Thr	0/45	1/105	0.716	0.397
23	3396T>C	Tyr>Tyr	0/45	1/105	0.716	0.397
24	3483G>A	Glu>Glu	0/45	1/105	0.716	0.397
25	3666G>A	Gly>Gly	1/45	0/105	2.424	0.12
26	3915G>A	Gly>Gly	0/45	1/105	0.716	0.397
27	3992C>T	Thr> Met	0/45	1/105	0.716	0.397
28	3593T>C	Val>Ala	0/45	1/105	0.716	0.397
29	3513C>T	Thr>Thr	1/45	1/105	0.354	0.552

^*^Statistically significant difference, *P* value < 0.05

**Table 4:** The percentages of men with total mitochondrial variants in (*ND1, ND2, ND5* and *ND6*) genes among controls and different asthenozoospermic groups

	Group 1 (*N* = 35)	Group 2 (N = 35)	Group 3 (N = 35)	Controls (*N* = 45)		
Gene	Number of males with total variants (percentage)	Number of males with total variants (percentage)	Number of males with total variants (percentage)	Number of males with total variants (percentage)	*X*^2^ (3, *N* = 150)	*P* value
ND1	28 (80.0%)	9 (25.7%)	6 (17.1%)	7 (15.6%)	47.9	0.0001
ND2	21 (60.0%)	11 (31.4%)	8 (22.9%)	6 (13.3%)	21.5	0.0008
ND5	32 (91.4%)	27 (77.1%)	15 (42.9%)	8 (17.8%)	52.9	0.0001
ND6	16 (45.7%)	11 (31.4%)	6 (17.1%)	6 (13.3%)	12.8	0.0051

A total of 21 nucleotide substitutions in the *ND2* gene were identified; six of them were missense, and 15 were synonymous (Table 5). The percentages of men with total variants in the *ND2* gene among groups 1, 2, 3, and in the control were 60%, 31.4%, 22.9%, and 13.3%, respectively, *P* = 0.0008 (Table 4). However, none of these variants were significantly different between the 2 groups (*P* value > 0.05).

**Table 5:** The mtDNA variants identified in the *ND2* gene

Serial number	MtDNA variant	Amino acid change	Frequency of variant in control	Frequency of variant in asthenozoospermia	G test	*P* value
1	4769A>G	Met>Met	2/45	12/105	2.061	0.151
2	4917A>G	Asn>Asp	1/45	11/105	3.601	0.058
3	4733T>C	Asn>Asn	0/45	1/105	0.716	0.397
4	4967T>C	Ser>Ser	0/45	1/105	0.716	0.397
5	4973T>C	Gly>Gly	0/45	2/105	1.438	0.23
6	4991G>A	Gln>Gln	1/45	4/105	0.266	0.606
7	5178C>A	Leu>Met	0/45	2/105	1.438	0.23
8	4646T>C	Tyr>Tyr	0/45	1/105	0.716	0.397
9	5302T>C	Ile>Thr	0/45	1/105	0.716	0.397
10	5417G>A	Gln>Gln	0/45	1/105	0.716	0.397
11	5237G>A	Pro>Pro	1/45	1/105	0.354	0.552
12	5331C>A	Leu>Ile	0/45	1/105	0.716	0.397
13	4561T>C	Val>Ala	0/45	1/105	0.716	0.397
14	4823T>C	Val>Val	1/45	4/105	0.266	0.606
15	5147G>A	Thr>Thr	0/45	1/105	0.716	0.397
16	4639T>C	Ile>Thr	0/45	2/105	1.438	0.23
17	4883C>T	Pro>Pro	0/45	1/105	0.716	0.397
18	4703T>C	Asn>Asn	1/45	0/105	2.424	0.12
19	5004T>C	Leu>Leu	1/45	0/105	2.424	0.12
20	4640C>A	Ile>Met	0/45	1/105	0.716	0.397
21	5048T>C	Val>Val	1/45	1/105	0.354	0.552

**Fig. 3:**
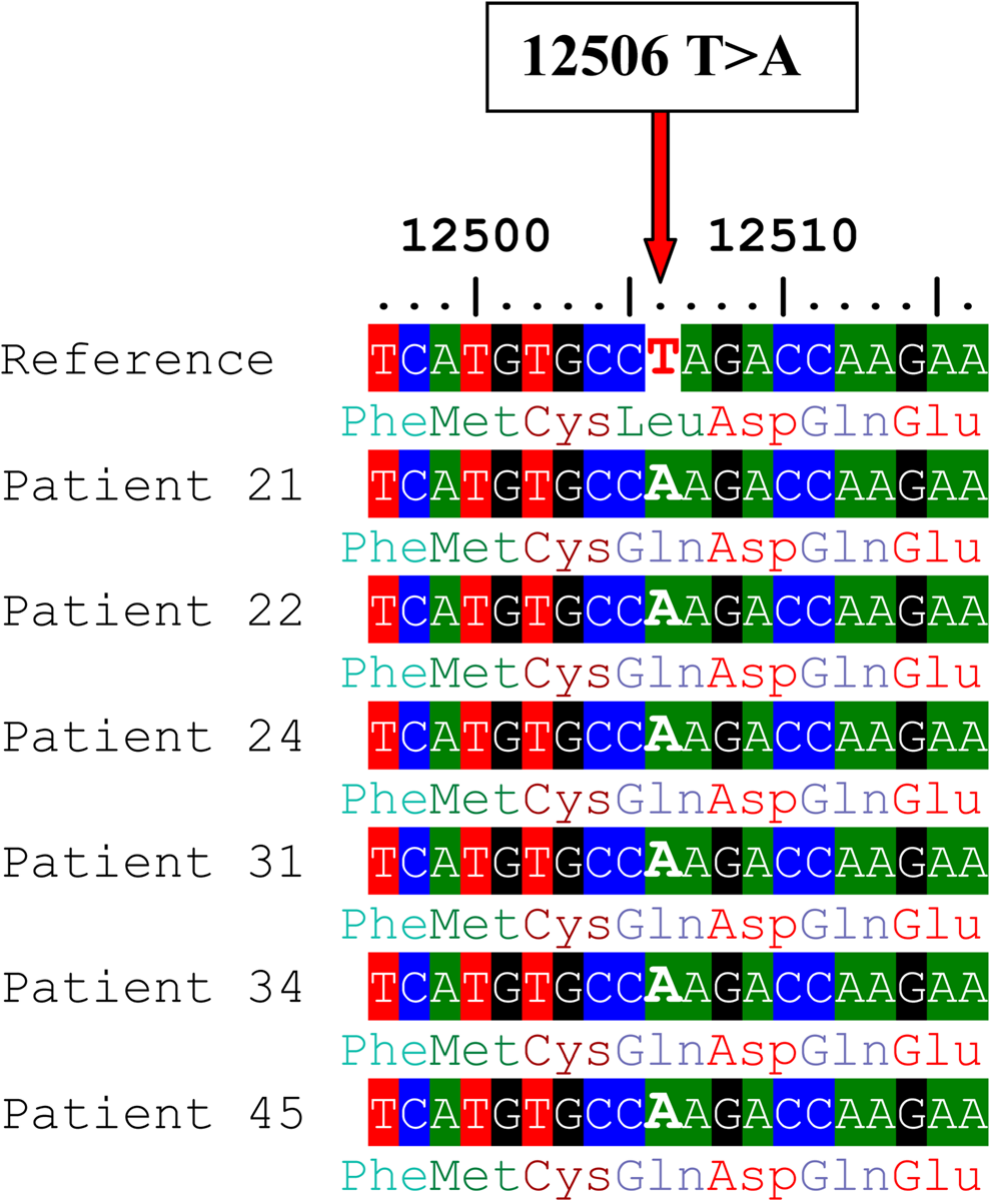
Alignment of the ND5 gene sequence for 6 patients showing the novel 12506T>A variant. The red arrow indicates the site of nucleotide substitution causing an amino acid replacement (Leu>Gln)

**Fig. 4:**
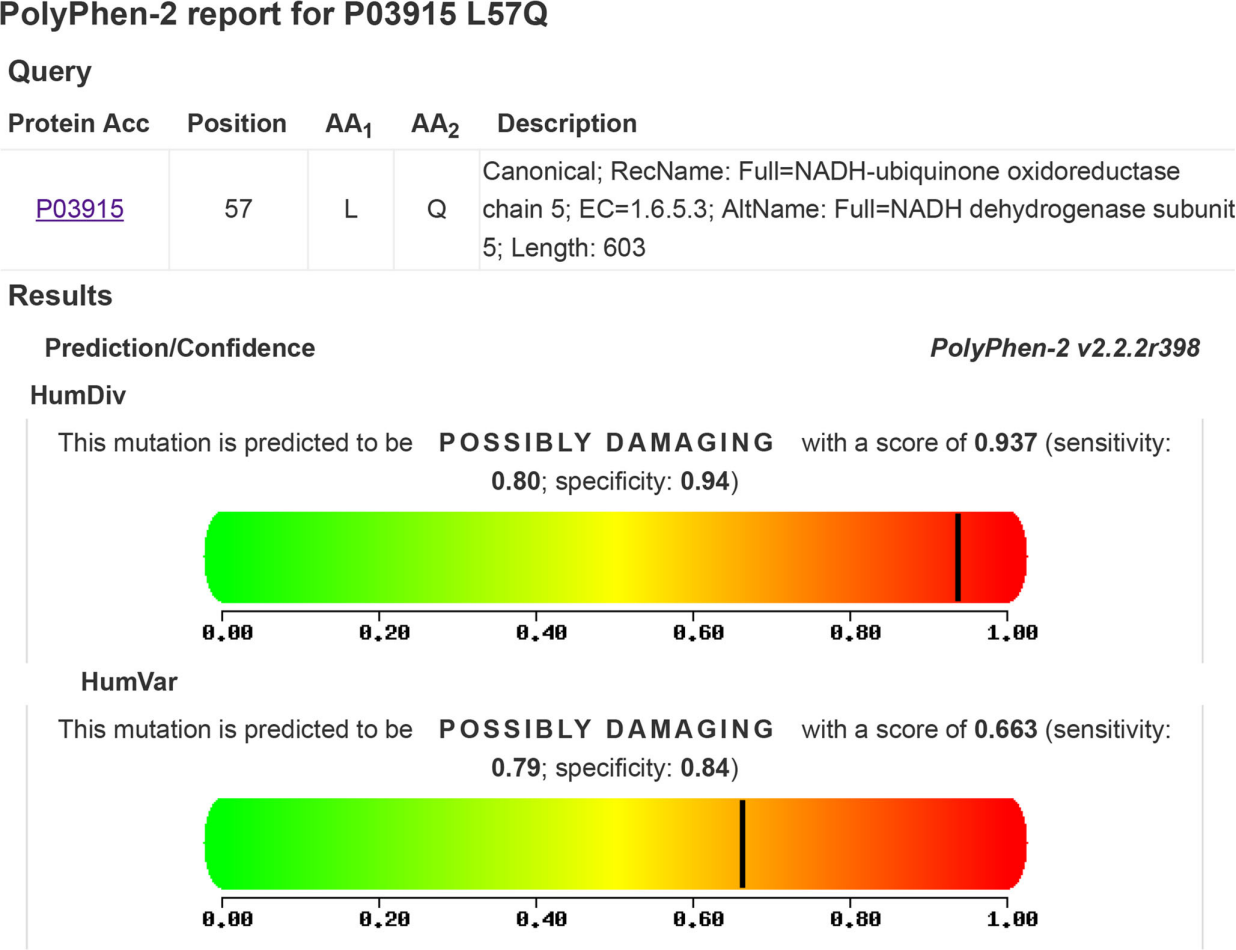
Prediction of the functional effect of 12506T>A substitution by Poly Phenyl-2 software

**Fig. 5:**
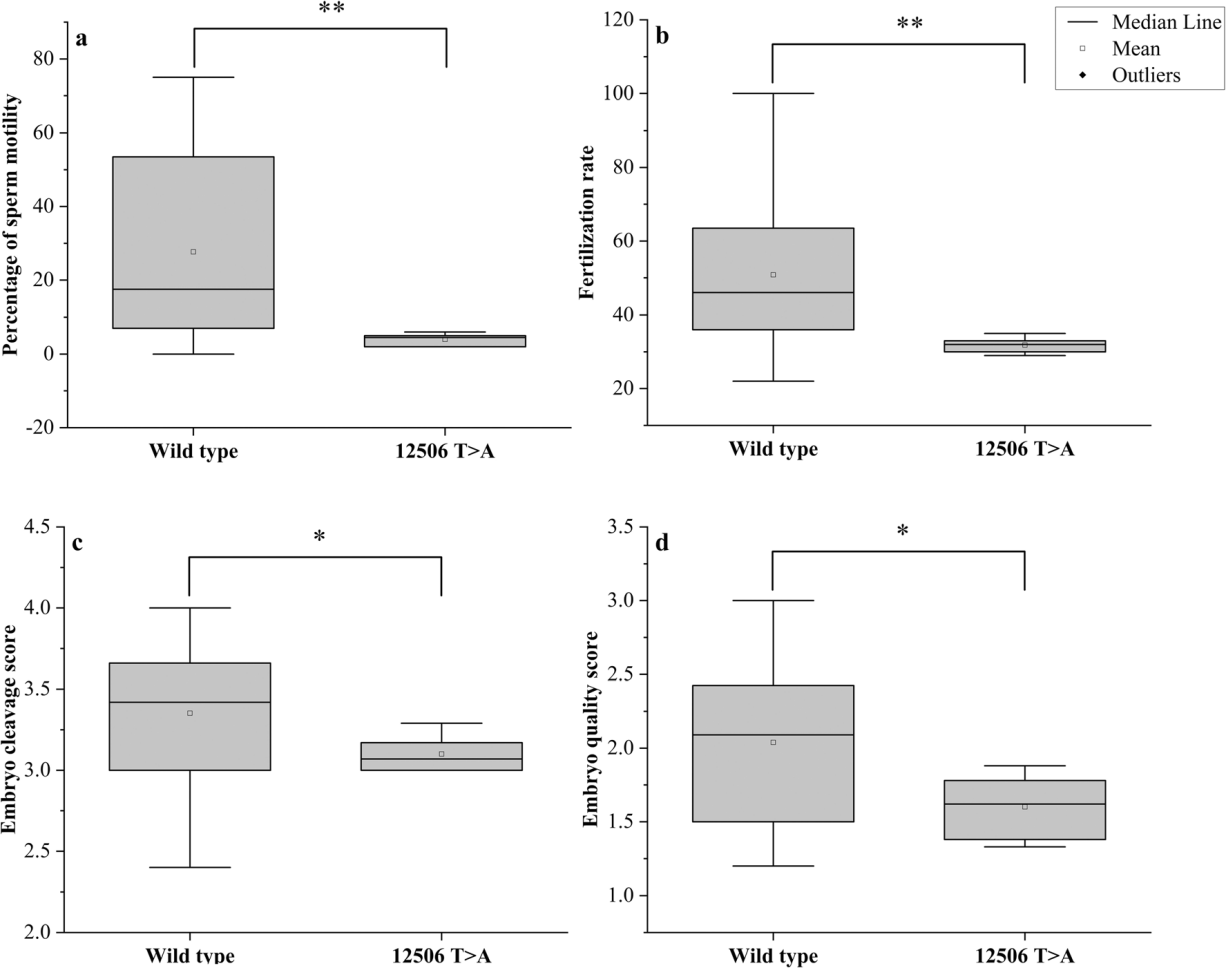
Box plots showing the differences between men with or without the 12506T>A. **a** Difference in sperm motility, **b** difference in fertilization rate, **c** difference in embryo cleavage score, and **d** difference in embryo quality score. Mann-Whitney *P* values for the differences in the medians were 0.009, 0.001, 0.044, and 0.028, respectively. *P < 0.05, **P < 0.01

A total of 19 missense variants and 37 synonymous variants were identified in the *ND5* gene (Table 6). The percentages of men with total variants in the *ND5* gene among groups 1, 2, 3, and the control were 91.4%, 77.1%, 42.9%, and 17.8%, respectively, *P* = 0.0001(Table 4). All of these variants had been previously reported in the NCBI (https://www.ncbi.nlm.nih.gov/) and in the human mitochondrial DNA database (www.mitomap.org), except for a novel variant at the locus 12506. This variant was identified in 6 asthenozoospermic patients (all were heteroplasmic), also the percentages of men with 12506T>A among groups 1, 2 and were 14.3%, 2.9% respectively, *P* = 0.0001 while it was not found among group 3 and the control. We reported the nucleotide sequences of 6 patients (BankIt2363991 ND5_21, BankIt2363991 ND5_22, BankIt2363991 ND5_24, BankIt2363991 ND5_31, BankIt2363991 ND5_34 and BankIt2363991 ND5_45) to the GenBank and the following accession numbers were given, respectively (MT742299, MT742300, MT742301, MT742302, MT742303 and MT742304). The novel SNP 12506T>A is a missense variant that replaced Gln by Leu (Fig. 3). This variant is significantly different between cases and controls (*P* = 0.036) and is predicted to be probably damaging according to the Poly Phenyl-2 and ACMG (score 0.663, sensitivity 0.79; specificity 0.84) (Fig. 4). The medians of sperm motility (wild type (17.5 ± 24.93), 12506T>A (4.5 ± 24.93), *P* = 0.009), fertilization rate (wild type (46 ± 17.8), 12506T>A (32 ± 2.13), *P* = 0.001), embryo cleavage score (wild type (3.42 ± 0.37), 12506T>A (3.07 ± 0.12), *P* = 0.044), embryo quality score (wild type (2.09 ± 0.49), 12506T>A (1.62 ± 0.22), *P* = 0.028) (Fig. 5).

**Table 6:** The mtDNA variants identified in the *ND5* gene

Serial number	MtDNA variant	Amino acid change	Frequency of variant in control	Frequency of variant in asthenozoospermia	G test	*P* value
1	13708G>A^*^	Ala>Thr	1/45	15/105	6.131	0.013*
2	13879T>C	Ser>Pro	0/45	1/105	0.716	0.397
3	13965T>C	Leu>Leu	0/45	1/105	0.716	0.397
4	13966A>G	Thr>Ala	0/45	1/105	0.716	0.397
5	13967C>T	Thr>Met	0/45	1/105	0.716	0.397
6	13928G>C	Ser>Asn	0/45	1/105	0.716	0.397
7	13734T>C	Phe>Phe	0/45	1/105	0.716	0.397
8	14040G>A	Gln>Gln	0/45	4/105	2.9	0.089
9	14070A>G	Ser>Ser	1/45	3/105	0.051	0.822
10	13650C>T	Pro>Pro	0/45	3/105	2.166	0.141
11	13752T>C	Ile>Ile	0/45	1/105	0.716	0.397
12	13803A>G	Thr>Thr	0/45	2/105	1.438	0.23
13	14059A>G	Ile>Val	0/45	1/105	0.716	0.397
14	13780A>G	Ile>Val	0/45	1/105	0.716	0.397
15	14053A>G	Thr>Ala	0/45	1/105	0.716	0.397
16	14110T>C	Phe>Leu	1/45	1/105	0.354	0.552
17	13762T>G	Ser>Ala	0/45	2/105	1.438	0.23
18	12372G>A	Leu>Leu	6/45	18/105	0.35	0.554
19	12705C>T	Ile>Ile	0/45	14/105	10.593	0.001
20	12850A>G	Ile>Val	0/45	3/105	2.166	0.141
21	12822A>G	Ala>Ala	0/45	4/105	2.9	0.089
22	12406G>A	Val>Ile	0/45	1/105	0.716	0.397
23	13722A>G	Leu>Leu	1/45	1/105	0.354	0.552
24	12346C>T	His>Tyr	0/45	2/105	1.438	0.23
25	12403C>T	Leu>Phe	0/45	2/105	1.438	0.23
26	12414T>C	Pro>Pro	0/45	2/105	1.438	0.23
27	12612A>G	Val>Val	0/45	8/105	5.898	0.015
28	12501G>A	Met>Met	2/45	3/105	0.234	0.629
29	12693A>G	Lys>Lys	0/45	1/105	0.716	0.397
30	12950A>G	Asn>Thr	0/45	1/105	0.716	0.397
31	12408T>C	Val>Val	0/45	1/105	0.716	0.397
32	13368G>A	Gly>Gly	1/45	13/105	4.83	0.028
33	13020T>C	Gly>Gly	0/45	1/105	0.716	0.397
34	13215T>C	Leu>Leu	0/45	1/105	0.716	0.397
35	13702C>G	Arg>Gly	0/45	1/105	0.716	0.397
36	13392T>C	Asn>Asn	0/45	2/105	1.438	0.23
37	13104A>G	Gly>Gly	1/45	5/105	0.589	0.443
38	13422A>G	Leu>Leu	0/45	2/105	1.438	0.23
39	13145G>A	Ser>Asn	1/45	2/105	0.016	0.9
40	13326T>C	Cys>Cys	0/45	1/105	0.716	0.397
41	13188C>T	Thr>Thr	0/45	1/105	0.716	0.397
42	13590G>A	Leu>Leu	0/45	2/105	1.438	0.23
43	13650C>T	Pro>Pro	0/45	2/105	1.438	0.23
44	13188C>T	Thr>Thr	0/45	1/105	0.716	0.397
45	13780A>G	Ile>Val	1/45	0/105	2.424	0.12
46	13981C>T	Pro>Ser	0/45	1/105	0.716	0.397
47	14025T>C	Pro>Pro	0/45	1/105	0.716	0.397
48	14034T>C	Ile>Ile	0/45	1/105	0.716	0.397
49	12630G>A	Trp>Trp	0/45	1/105	0.716	0.397
50	12654A>G	Trp>Trp	0/45	1/105	0.716	0.397
51	12681T>C	Asn>Asn	0/45	1/105	0.716	0.397
52	13542A>G	Ser>Ser	0/45	1/105	0.716	0.397
53	13617T>C	Ile>Ile	0/45	1/105	0.716	0.397
54	13821C>T	Phe>Phe	0/45	3/105	2.166	0.141
55	12506T>A*	Leu>Gln	0/45	6/105	4.386	0.036*
56	12879T>C	Gly>Gly	0/45	3/105	2.166	0.141

^*^Statistically significant difference, *P* value <0.05

**Fig. 6:**
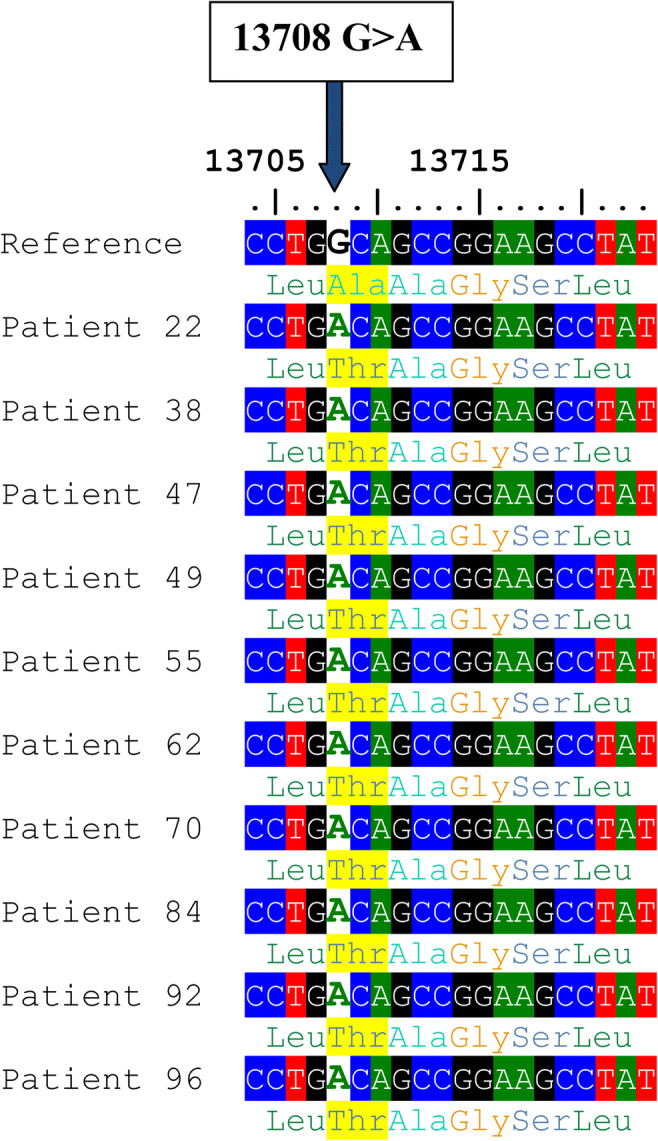
Alignment of the *ND5* gene sequence for 10 patients showing 13708G>A variant. The blue arrow indicates the site of nucleotide substitution, and the highlighted yellow colour indicates the amino acid replacement (Ala>Thr)

**Fig. 7:**
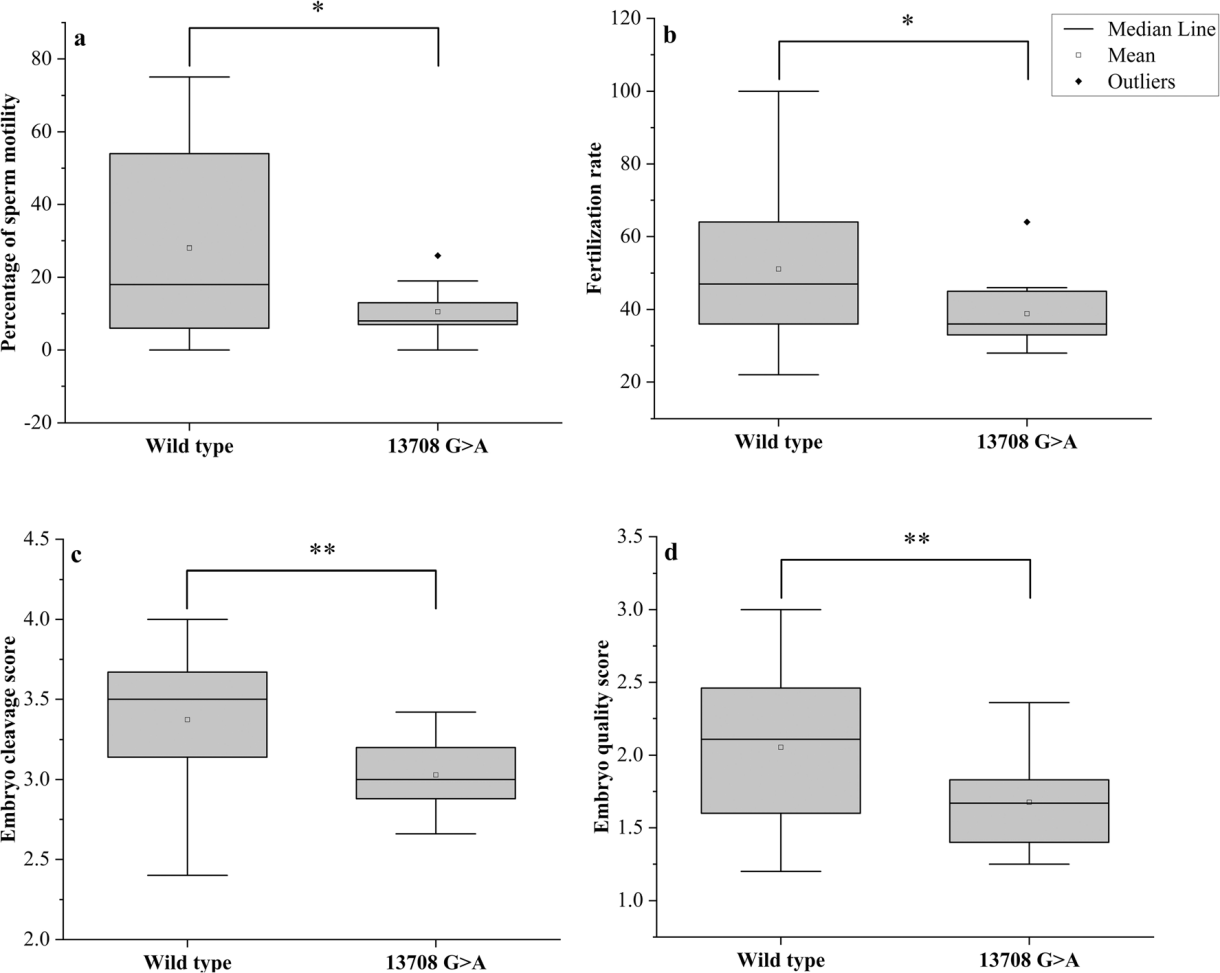
Box plots showing the differences between men with or without the 13708G>A. **a** Difference in sperm motility, **b** difference in fertilization rate, **c** difference in embryo cleavage score, and **d** difference in embryo quality score. Mann-Whitney *P* values for the differences in the medians were 0.043, 0.017, 0.001, and 0.007, respectively. *P < 0.05, **P < 0.01

Another variant 13708G>A (rs28359178) in the *ND5* gene was significantly different between cases and controls (*P* = 0.013), causing Ala>Thr amino acid substitution (Fig. 6). However, this variant is predicted to be benign according to the Poly Phenyl-2 and ACMG criteria (score 0.001, sensitivity 0.99; specificity 0.15). The 13708G>A variant was identified among 14 asthenozoospermic men only—all were homoplasmic—while it was not found among control group. The percentages of men with 13708G>A among groups 1, 2, and 3 were 17.1%, 14.3%, and 8.6%, *P* = 0.0012. The medians of sperm motility (wild type (18 ± 25.46), 13708G>A (8 ± 7.13), *P* = 0.043), fertilization rate (wild type (47 ± 18.09), 13708G>A (36 ± 9.55), *P* = 0.017), embryo cleavage score (wild type (3.5 ± 0.36), 13708G>A (3.0 ± 0.24), *P* = 0.001), embryo quality score (wild type (2.11 ± 0.49), 13708G>A 1.67 ± 0.35), *P* = 0.007) (Fig. 7).

A total of 15 nucleotide substitutions was identified in the *ND6* gene; two of them were missense, while the rest were synonymous variants (Table 7). The percentages of men with total variants in the ND6 gene among groups 1, 2, 3, and control were 45.7%, 31.4%, 17.1%, and 13.3%, respectively, *P* = 0.0051(Table 4). However, none of these variants were significantly different between the two groups.

**Table 7:** The mtDNA variants identified in the *ND6* gene

Serial number	MtDNA variant	Amino acid change	Frequency of variant in control	Frequency of variant in asthenozoospermia	G test	*P* value
1	14167C>T	Glu>Glu	0/45	4/105	2.9	0.089
2	14179A>G	Tyr>Tyr	1/45	2/105	0.016	0.9
3	14182T>C	Val>Val	0/45	3/105	2.166	0.141
4	14233A>G	Asp>Asp	2/45	12/105	2.061	0.151
5	14323G>A	Asp>Asp	0/45	3/105	2.166	0.141
6	14364G>A	Leu>Leu	1/45	7/105	1.438	0.23
7	14560G>A	Val>Val	1/45	1/105	0.354	0.552
8	14620C>T	Gly>Gly	0/45	1/105	0.716	0.397
9	14470T>C	Gly>Gly	0/45	1/105	0.716	0.397
10	14566A>G	Gly>Gly	0/45	2/105	1.438	0.23
11	14569G>A	Ser>Ser	0/45	2/105	1.438	0.23
12	14178T>C	Ile>Val	0/45	2/105	1.438	0.23
13	14180T>C	Tyr>Cys	0/45	2/105	1.438	0.23
14	14212T>C	Val>Val	1/45	2/105	0.016	0.9
15	14305G>A	Ser>Ser	1/45	1/105	0.354	0.552

**Fig. 8:**
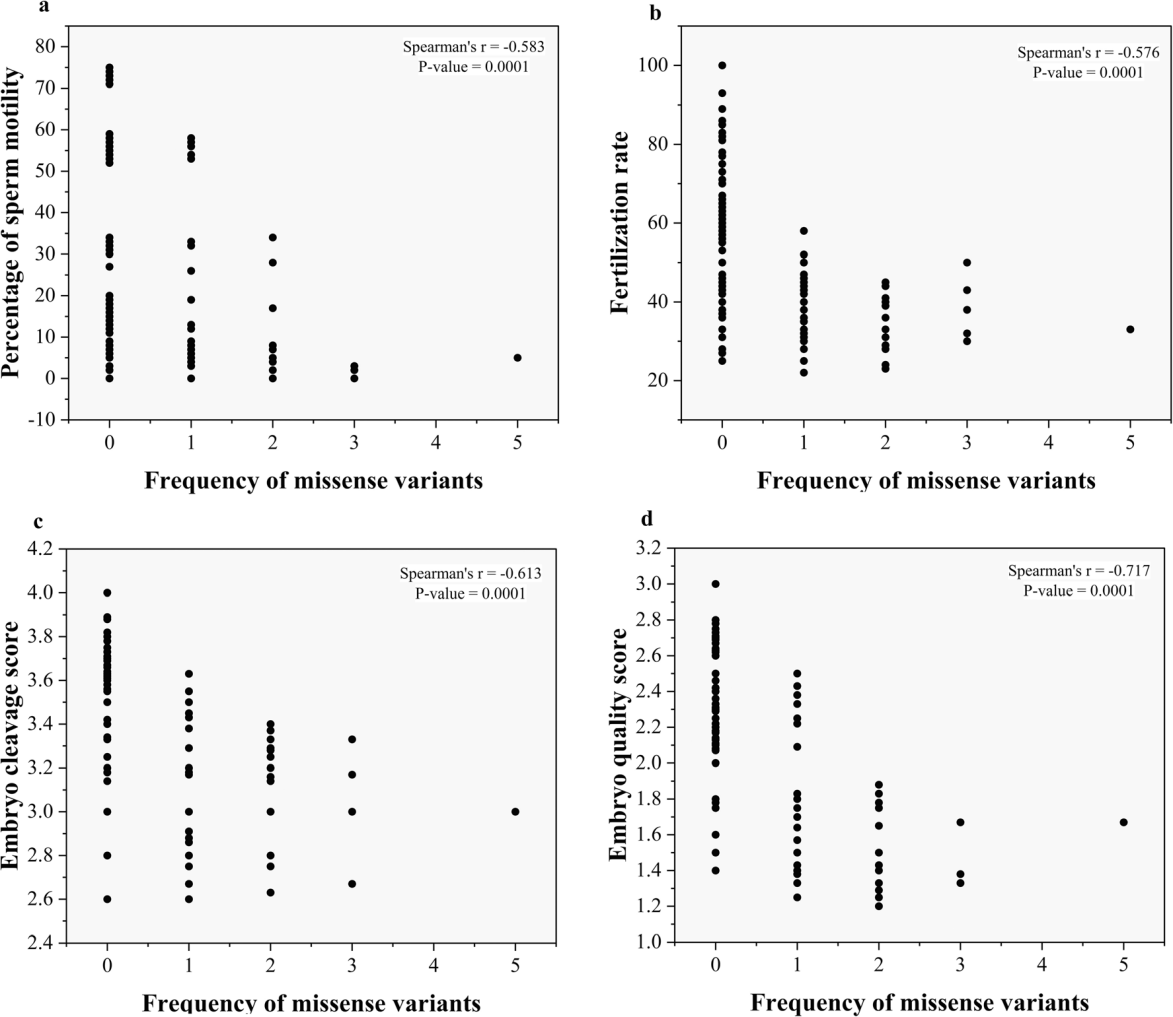
Scatter plots of frequency of missense variants with sperm motility and ICSI outcomes. **a** Frequency of missense variants with sperm motility (*r* = − 0.583, *P* = 0.0001). **b** Frequency of missense variants with fertilization rate (*r* = − 0.576, *P* = 0.0001). **c** Frequency of missense variants with embryo cleavage score(*r* = − 0.613, P = 0.0001). **d** Frequency of missense variants with embryo quality score (*r* = − 0.717, *P* = 0.0001)

**Fig. 9:**
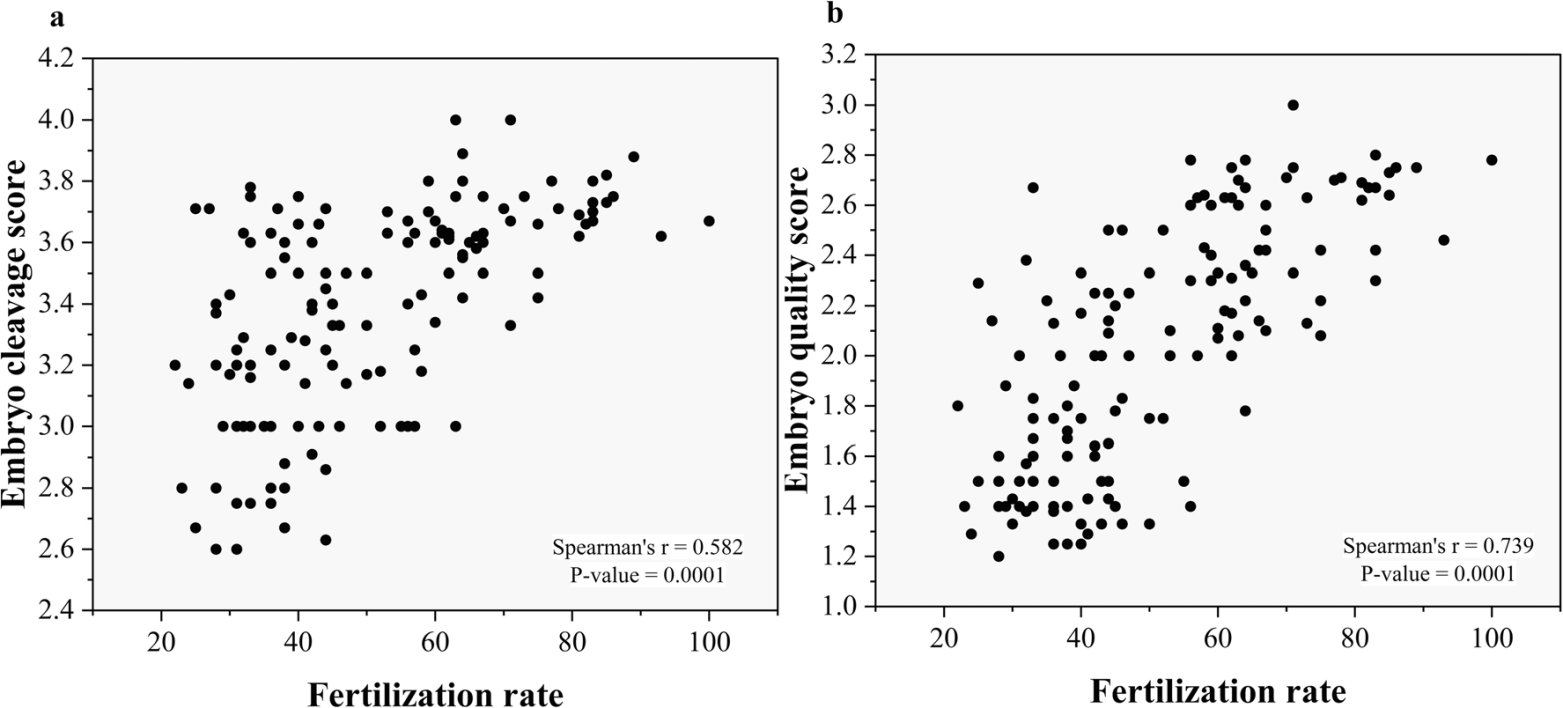
**a** Scatter plots of fertilization rate and embryo cleavage score showing a positive correlation, *r* = 0.582, *P* = 0.0001. **b** Scatter plots of fertilization rate and embryo quality score showing a positive correlation, *r* = 0.739, *P* = 0.0001

**Fig. 10:**
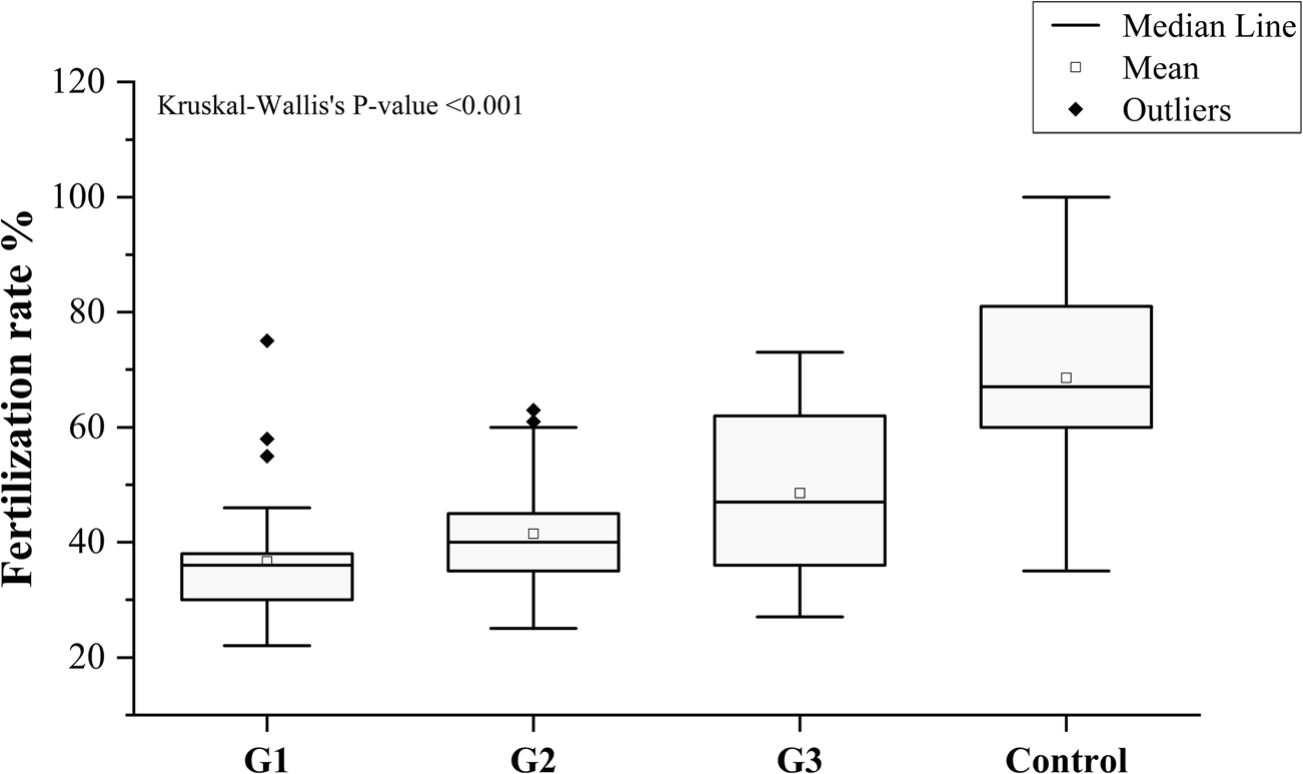
Fertilization rate among groups 1, 2, 3, and control. Kruskal-Wallis’s *P* value was included. Mann-Whitney *P* values for the differences in the medians between all groups < 0.001

The frequency of all missense variants had a significant inverse relationship with sperm motility, fertilization rate, embryo cleavage score, and embryo cleavage score (*r* = − 0.583, *P* = 0.0001), (*r* = − 0.576, *P* = 0.0001), (*r* = − 0.613, *P* = 0.0001), and (*r* = − 0.717, *P* = 0.0001), respectively (Fig. 8). The fertilization rate had a significant positive relationship with the embryo cleavage score and the embryo quality score (*r* = 0.582 and *P* = 0.0001) (*r* = 0.739 and *P* = 0.0001), respectively (Fig. 9). The median fertilization rates were as follows: (G1 (36 ± 1.86), G2 (40 ± 1.63), G3 (47 ± 13.41), control (67 ± 14.69), *P* < 0.001) as illustrated in (Fig. 10).

**Fig. 11:**
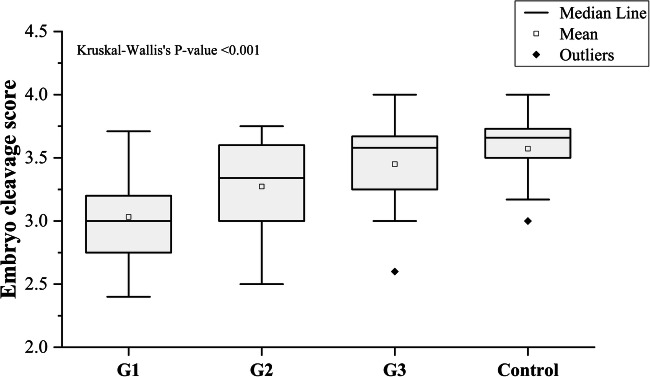
Embryo cleavage score among groups 1, 2, 3, and control. Kruskal-Wallis’s *P* value was included. Mann-Whitney *P* values for the differences in the medians between all groups < 0.001

**Fig. 12:**
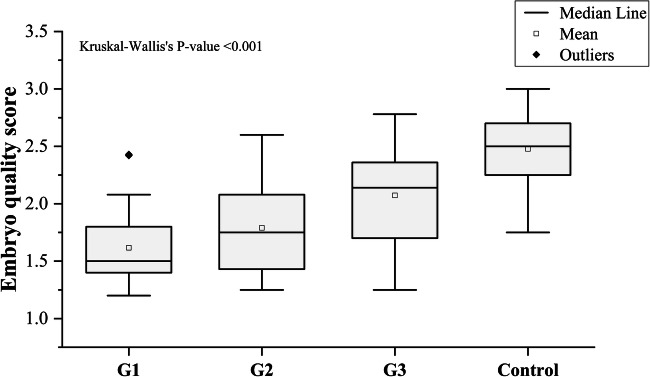
Embryo quality score among the studied groups. Kruskal-Wallis’s *P* value was included, Mann-Whitney *P* values for the differences in the medians between all groups < 0.001

The average embryo quality score and cleavage score among asthenozoospermic patients were 1.83 and 3.24, respectively, compared to 2.47 and 3.58, respectively, in normozoospermic ones. A significant difference in the embryo cleavage score was detected between patients and controls (*P* = 0.0001). We also found a significant difference between the embryo quality score in the two categories (*P* = 0.0001). The median of the embryo cleavage scores were statistically different between the different groups: (G1 (3 ± 0.31), G2 (3.34 ± 0.36), G3 (3.58 ± 0.31), control (3.66 ± 0.25), *P* < 0.001), as illustrated in (Fig. 11). The median of the embryo quality scores were also statistically different: (G1 (1.5 ± 0.31), G2 (1.75 ± 0.39), G3 (2.14 ± 0.44), control (2.5 ± 0.27), *P* < 0.001) (Fig. 12).

## Discussion

In the present study, we found three missense variants correlated inversely with sperm motility and ICSI outcomes. Two variants, namely, 13708G>A (rs28359178) in the *ND5* gene and 4216T>C (rs1599988) in the *ND1*, were previously reported in the NCBI (https://www.ncbi.nlm.nih.gov/) and human mitochondrial DNA database (www.mitomap.org), while we identified 12506T>A as a novel variant in the *ND5* gene.

The variants detected in the present study have not been linked to asthenozoospermia before. However, a previous study showed that the rate of 4216T>C variant among diabetic patients was higher than in the controls and was statistically associated with diabetes mellitus type 2 [26]. Another study found an interesting male-specific association between the 4216T>C variant and the rate of infection leading to complicated sepsis and death [27].

Similarly, the 13708G>A variant has been linked to several clinical manifestations. It has been shown that the 13708G>A variant increases the susceptibility to multiple sclerosis [28] and found to enhance the expression of Leber hereditary optic neuropathy (LHON) disease in another study [29]. Furthermore, 13708G>A variant was found to increase the risk for Alzheimer’s disease, specifically among male patients [30].

Our results showed a negative correlation between sperm motility and the frequency of total variants, where the highest frequency of mitochondrial variants in the four genes was among group one (asthenozoospermic patients with 0 to 5% sperm motility), while the lowest frequency was among group three (asthenozoospermic patients with 16 to 35% sperm motility). The control group with normozoospermia (sperm motility between 50 and 75%) showed a lower frequency of mitochondrial variants, compared to all patients’ groups. A previous study reported that 9055G>A in the ATPase gene and 11719G>A in *ND4* were associated with poor semen quality [18]. Another variant 11994C>T in *ND4* gene was negatively correlated with oligoasthenozoosperima [31].

It is known that sperms require ATP for flagellar movement, and this depends on OxPhos to provide their energy requirements [3]. Here, we identified three missense variants with a significant association with sperm motility. These variants are located in the *ND1* and *ND5* genes, which are part of complex 1 [32]. Complex 1 plays a key role in OxPhos by receiving electrons from NADH, and the captured energy from these electrons is utilized to release protons to the intermembrane space, and these protons are used later to generate ATP [33]. Therefore, pathogenic variants in the *ND* genes are expected to affect complex 1 activity, causing a deficiency in energy production, and this will negatively affect the sperm motility [7].

Our results demonstrated that the ICSI outcomes were correlated inversely with the load of mtDNA variants, while it had a strong positive correlation with the percentage of sperm motility, where the fertilization rate was lower among group one compared to group three. Embryo quality, including embryo cleavage score and embryo quality score, were positively correlated with sperm motility. Embryos among group one had the lowest quality scores, while the best embryo quality was among controls. Furthermore, the three variants (13708G>A, 4216T>C, and 12506T>A) were negatively correlated with ICSI outcomes. Patients with those variants showed lower fertilization rate and embryo quality scores compared to other men without these variants.

Interestingly, our findings agree with the results of a previous study where they reported that mtDNA mutations reduce motility and negatively affect the fertilization rate [34]. Our results also agree with a previous study that identified statistical associations between seven variants in the *ND5* gene and fertilization failure [35]. In the present study, we determined that the embryo quality score has a negative correlation with the frequency of total mtDNA variation. Embryos with no or low mtDNA variants have a high probability of reaching grade A on day 3. A recent study has reported that mtDNA variations were correlated negatively with embryo grading, and embryo quality at the blastocyst stage was correlated positively with sperm motility [20].

However, our results disagree with previous findings where no significant difference between the frequency of variants in mtDNA among teratozoospermia and asthenozoospermia was found [36]. However, these results do not provide conclusive evidence against the role of mtDNA in sperm motility, because of the small sample (43 samples only). The contradiction between the results of this study and other studies may be attributed to population variation. In a previous study, they found that the frequency of mtDNA SNPs varies between African American, European and Asian populations for the same mitochondrial disease [37]. Two independent studies on the association of the same SNP, namely 11994C>T, and oligoasthenozoosperima have reached different conclusions, where a strong association was found in India [31], while in the other study in Portugal no association was found [38]. The severity of the mitochondrial diseases depends on the level of heteroplasmy and the threshold value mutant mtDNA should pass in order to show pathogenic effects, which ranges between 60 and 80%, based on the types of mutations and cells [4].

We think that the criteria used in the current study for sample selection and sample grouping influenced the results obtained. We excluded many environmental factors that are known to affect sperm motility, such as smoking, varicocele, alcoholism, and men older than 40 years old. These factors have already been shown to affect sperm motility, and thus excluding them increases the probability of identifying the genetic etiology [39, 40]. Most of the previous studies, the patients’ grouping was done by including all sperm abnormalities together as one group, while in our study asthenozoospermic patients were divided into different categories, where the reduced sperm motility varied between 0 and 35%. Samples with sperm motility between 36 and 40% were excluded to stay far from the borderline of the normal percentage (40%) of total sperm motility recommended by the WHO [41]. We also relied on the percentage of total sperm motility rather than on the type of sperm motility, because, according to WHO, progressive motility (type A and type B) should be greater than 32%. So, as our samples were evaluated by different embryologists, it could be more reliable to depend on total sperm motility where it is easier to distinguish between motile and immotile sperm, rather than determining the specific type of sperm; this also decreases the number of individual errors among technicians.

Recent studies have provided evidence supporting the paternal transmission of mtDNA. Luo and co-workers presented a strong evidence for a bi-parental mtDNA inheritance, following the pattern of mitochondrial disease inheritance in three separate multi-generation families; they showed evidence of parental mtDNA transmission from father to offspring [15]. Ecker et al. found that sons who were born by ICSI shared the same SNPs in mitochondrial genes (*COX1*, *ND1*, *ND4*, and *ND5*) with their fathers; also, they found that the degree of similarity reached in some cases up to 99% of the paternal mtDNA [17]. Another study found that mtDNA myopathy can be transmitted from the father to the son by ICSI, and that spermatozoa mtDNA mutations were maintained in the embryo [42]. However, it is expected that the effect of paternal mtDNA variants will be diluted since the unfertilized oocyte contains around 150,000 copies of mtDNA, compared to sperm which contain around only 100 copies [43]. It remains to be determined how common paternal mtDNA inheritance is especially in ICSI settings, and the phenotypic consequences it may cause.

## Conclusions

We found that the frequencies of total mitochondrial variants in *ND1*, *ND2*, *ND5*, and *ND6* genes were negatively correlated with the percentages of sperm motility and ICSI outcomes. We also identified three variants, 13708G>A, 4216T>C, and 12506T>A, to be negatively correlated with sperm motility and ICSI outcomes. Future studies are needed to determine the functional consequences of the identified variants, and to understand the mechanism of how the fertilization rate is affected by sperm mtDNA, specifically in the early stage of embryo development. Despite the recent studies to identify the genetic basis of male infertility in Jordan [44–46], its cause remains unknown in a large number of cases. High-throughput genomic studies to identify the genetic etiology for infertility in Jordan should therefore be undertaken.

## Data Availability

Data are available upon request from the corresponding author.

## Abbreviations

ICSI: Intracytoplasmic sperm injection
MtDNA: Mitochondrial DNA
ROS: Reactive oxygen species
OxPhos: Oxidative phosphorylation
SNP: Single nucleotide polymorphism
IVF: In vitro fertilization
WHO: World Health Organization
HEPES: 4-(2-Hydroxyethyl)-1-piperazineethanesulfonic acid
PCR: Polymerase chain reaction
NCBI: National Centre of Biotechnology Information
ACMG: American College of Medical Genetics and Genomics
SQSTM1: Sequestosome 1
MDPs: Mitochondrial-derived peptides
UPR: Unfolded protein response
PR: Progressive motility
NP: Non-progressive motility
